# An unusual case of penetrating ocular trauma with metallic spoon

**DOI:** 10.4103/0301-4738.64128

**Published:** 2010

**Authors:** Gautam Bhaduri, Soumya Swarup Chattopadhyay, Rudra Prasad Ghosh, Kumar Saurabh, Mukesh Goyal

**Affiliations:** Regional Institute of Ophthalmology, Kolkata, West Bengal, India

**Keywords:** Blast, foreign body, injury, trauma

## Abstract

Ocular trauma is an important cause of vision loss. The agents incriminated in such injuries are diverse. We present a case of ocular trauma with a metallic spoon causing deep laceration of lid and temple region with sclerocorneal laceration. After assessment of the general condition and stabilization of the systemic parameters the operative procedure was undertaken on elective basis. Though the final visual outcome was not rewarding due to the severity of the injury, any potential hemostatic catastrophe was averted.

Ocular trauma is a leading cause of blindness.[1] The causative agents implicated in such cases are variable and related to the occupation and daily activities of the person. While there have been reports of blinding ocular trauma caused by common objects like wood pieces, pencil, firecrackers, there also have been reports of such trauma, caused by unusual objects like horse hoof,[2] paintball pellets[3] and grease from high hydraulic machinery.[4] We present an unusual case of blinding orbital trauma with sclerocorneal laceration by a metallic spoon.

## Case Report

A 30-year-old male patient presented to our emergency with bomb blast injury to his right eye. The incidence took place at a countryside liquor shop which allegedly housed a crude bomb-making unit. The spoons were being used to fill in the shell with explosive mixtures.

**Figure 1:**
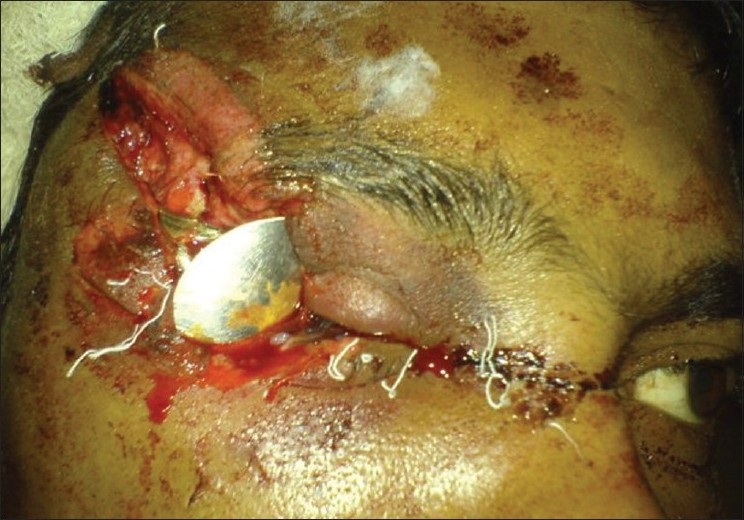
Metallic spoon impacted into right globe

**Figure 2:**
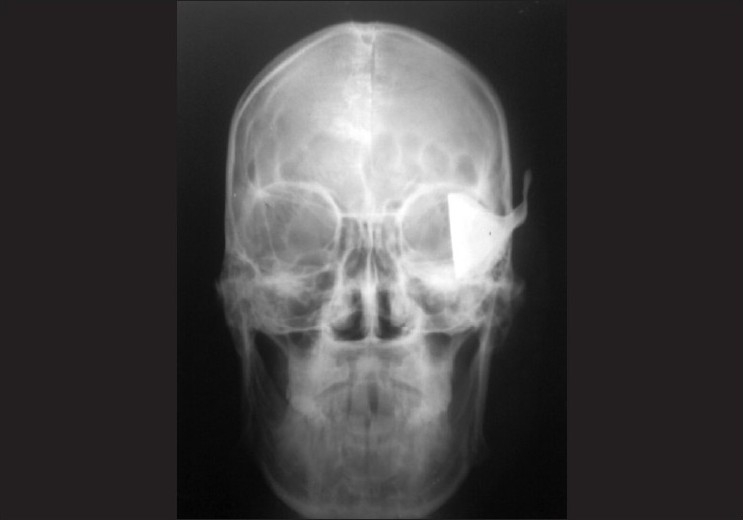
X-ray film showing handle of spoon in soft tissue with intact bony anatomy

Vision at presentation was reduced to light perception. Emergency room examination revealed a pulse rate of 68 per min, blood pressure 100/60 mm of Hg with Glassgow Coma Scale score of 13. A deep laceration was noted over his right temple which extended to the right upper eye lid and globe. Careful examination showed some metallic plate-like object impacted in his right eye from the temporal side [Fig. 1]. The lids were intact except at the temporal margin. Attempted ocular examination after prying the lids apart revealed deformed eyeball with metallic plate of the spoon securely fixed in the orbit with its handle in the laceration line at the temple. A sclerocorneal laceration was noted which extended from the temporal limbus to the medial canthus through the pupillary axis. There was extrusion of the ocular contents. An X-ray of the skull and orbit was obtained. The radiograph [Fig. 2] revealed that the handle of the steel spoon was lying deep in the laceration line on his temple with its plate entering into the globe through the right upper eye lid. The plate of the spoon was angulated at its junction with the handle. There was no fracture of the bony architecture along the course of soft tissue injury. The patient gave information that something had hit him from his right side at the time of explosion. After proper resuscitation with intravenous fluids and cross-matched blood transfusion, assessment of possible damage was done. The spoon was removed from his temple and orbit under general anesthesia the next morning. Sclerocorneal laceration was repaired with 6-0 Vicryl and 10-0 monofilament nylon. Upper lid laceration was repaired with the 6-0 Vicryl sutures after removal of residual infected ocular tissue. Patient was maintained on intravenous ceftriaxone (1 g every 12 h) and intravenous gentamicin (80 mg every 12 h) systemically along with topical fortified cefazolin (50 mg/ml) and fortified tobramycin (14 mg/ml). At one-week follow-up patient had visual acuity of hand movement close to face. Ultrasonography B scan at one week revealed vitreous hemorrhage and total retinal detachment. Pars plana vitrectomy with retinal reattachment was contemplated, however, poor wound integrity led us to postpone the surgery to a later date. Resolving vitreous hemorrhage was noted at next follow-up visit at one month. Visual acuity persisted at hand movement close to face mainly due to corneal opacity which precluded fundoscopy.

## Discussion

Penetrating ocular trauma is a leading cause of unilateral blindness.[1] Different modes and settings of the trauma may necessitate a change in the approach to the management of such cases. Like in our case, a prompt use of antibiotics and intraoperative hemostasis were the cornerstone of the successful revival of the patient.

The impacted foreign body in the orbit may be organic or inert. Organic foreign bodies like wood need to be removed at the earliest due to the associated high risk of infection. Inert materials like glass, plastic or steel are associated with lesser risk of infection and a decision to remove them should be based on factors like site of impingement, size of the foreign body, potential of secondary injuries and hemostasis.[5] The physical characteristics of the foreign body like mass and shape are also of prognostic importance. Woodcock *et al*.[6] from UK had found that foreign bodies of greater mass were associated with worse visual outcome. Lid laceration and adnexal injuries have been found to be among other the factors associated with eventual enucleation of injured the eye.[7]

X-ray and computed tomography scan remain the investigations of choice for ocular or orbital trauma cases. The decision to operate should be based upon proper evaluation of the systemic condition of the patient. Many reports have found that deferral of surgical procedure until stabilization of patient did not influence the final visual outcome.[7,8] In the present case operative procedures were undertaken the next morning after overnight resuscitation. The decision to defer the operation was based on the fact that the foreign body was non-reactive and preparation for any possible intraoperative hemorrhage was deemed necessary before surgery. In extreme cases Bhaduri *et al*.[9] had reported removal of a wooden foreign body from the anterior chamber of an eye after 25 years of initial injury with good postoperative vision.

In our patient the systemic condition at presentation was sufficient enough for us to postpone the surgical removal of the spoon till the next scheduled operative session though the final visual outcome was not rewarding.
